# Current Status of the Screening of Chlamydia trachomatis Infection Among Japanese Pregnant Women

**DOI:** 10.14740/jocmr2137w

**Published:** 2015-05-08

**Authors:** Shunji Suzuki, Masanobu Tanaka, Hideo Matsuda, Yuki Tsukahara, Yasushi Kuribayashi, Akihiko Sekizawa, Ryoichiro Miyazaki, Osamu Nishii, Akihito Nakai, Nobuko Mizutani, Yoshiaki Kumamoto, Katsuyuki Kinoshita

**Affiliations:** a Japan Association of Obstetricians and Gynecologists, Japan; b Japanese Foundation for Sexual Health Medicine, Japan

## To the Editor

Chlamydia trachomatis (CT) infection in pregnancy can cause maternal disease, adverse pregnancy outcomes, and neonatal disease [1-6]. In general, approximately 80% of CT infected women are asymptomatic or minimally symptomatic. Therefore, screening is the only means to effectively identify infections [2]. In Japan, pregnant women are routinely tested for CT with the Japanese public funds. According to the guidelines for obstetrical practice in Japan [7], Japanese obstetricians must provide a test for the detection of CT for the prevention of neonatal CT infection and diagnose urogenital CT infection when CT is detected using polymerase chain reaction (PCR), strand displacement amplification, transcription mediated amplification, an enzyme immunoassay, or culture methods in specimens obtained from the uterine cervix (CT nucleic acid detection tests). However, CT antibody detection by IgA tests has been substituted for these methods by some of obstetricians.

On October 2014, we requested 2,544 obstetrical facilities that are members of Japan Association of Obstetricians and Gynecologists (JAOG) to provide information of CT screening tests in pregnant women between October 2013 and March 2014. A total of 1,644 (64.6%) of 2,544 obstetrical facilities responded with possible statistical analysis information on a total of 328,788 women, accounting for approximately 65% of all deliveries that occurred in Japan during the study period. Of the 1,644 obstetrical facilities, CT nucleic acid detection PCR tests, CT nucleic acid detection tests except PCR and CT antibody detection tests were performed in 1,221 (74.3%), 408 (24.8%) and 15 (0.9%) facilities, respectively.

Table 1 shows the maternal age distribution under the three CT screening tests (CT nucleic acid detection tests with and without PCR and CT antibody detection tests). There were no significant differences in the maternal age distribution among the three CT screening tests groups.

**Table 1:** Maternal Age Distribution Under the Three Chlamydia trachomatis (by Nucleic Acid Detection Tests With and Without Polymerase Chain Reaction and Antibody Detection Tests)

Maternal age (years)	PCR test		Nuclei acid tests except PCR		Antibody tests	
	Number	(%)	Number	(%)	Number	(%)
19	5,370	2.1	1,675	2.2	60	2.1
20 - 24	26,049	10.4	8,224	10.9	374	12.8
25 - 29	65,503	26.1	20,814	27.5	792	27.1
30 - 34	82,194	32.8	25,131	33.2	1,015	34.7
35 - 39	51,937	20.7	15,217	20.1	573	19.6
40	13,190	5.3	3,467	4.6	108	3.7
Total	250,571	100	75,795	100	2,922	100

PCR: polymerase chain reaction.

Table 2 shows the results of CT screening tests (CT nucleic acid detection tests with and without PCR and CT antibody detection tests) of the study population by age. There were no significant differences in the rate of positive CT between the two groups of CT nucleic acid detection screening tests with and without PCR. However, the positive rate by the CT antibody detection tests was significantly higher than those by the two CT nucleic acid detection tests with and without PCR (P < 0.01 by X^2^ test).

**Table 2:** Prevalence of Chlamydia trachomatis Screening Tests (by Nucleic Acid Detection Tests With and Without Polymerase Chain Reaction and Antibody Detection Tests) of the Study Population by Age

Maternal age (years)	PCR test		Nuclei acid tests except PCR		Antibody tests	
	Positive	Positive rate (%)	Positive	Positive rate (%)	Positive	Positive rate (%)
19	854/5,370	15.9	272/1,675	16.2	15/60	25^#^
20 - 24	1,953/26,049	7.5	556/8,224	8.0	66/374	18.2*
25 - 29	1,533/65,503	2.3	462/20,814	2.2	96/792	12.1*
30 - 34	965/82,194	1.2	347/25,131	1.4	99/1,015	9.8*
35 - 39	408/51,937	0.8	136/15,217	0.9	57/573	9.9*
40	129/13,190	1.0	31/3,467	0.9	15/108	13.4*
Total	5,880/250,571	2.3	18,07/75,795	2.4	348/2,922	11.9*

PCR: polymerase chain reaction. ^#^P = 0.055 vs. values with CT nucleic acid detection PCR tests by the X^2^ test. *P < 0.01 vs. values with CT nucleic acid detection PCR tests by the X^2^ test.

The current CT prevalence rate in the Japanese pregnant women with even higher rates among pregnant teenagers is almost compatible with some previous observations in other countries [1, 8-11]. In some studies reported age-based estimates, younger participants had higher prevalence estimates than older participants associated with the cervical biological immaturity [12]. The previous reports showed that CT infection rates are highest among those < 25 years and are also consistent with sexual behavior data which show that numbers of sexual partners are highest in these younger age groups [13].

The data revealed that the rates of CT detection by the both CT nucleic acid detection tests with and without PCR differ significantly from those by the CT antibody detection tests. In an earlier study by Weill et al [14], sensitivity and/or specificity of CT antibody detection tests against CT nucleic acid detection tests have been reported to be not high enough. In an earlier study in Japan [15], it has been observed that CT antibodies will not be detected if CT infection is confined in the columnar epithelium of the uterine cervix. In addition, in our preliminary study [16], we found two cases with positive CT nucleic acid amplification tests in 97 pregnant women with negative CT antibody detection tests (2.1%). According to the guidelines for obstetrical practice in Japan [7], treatment with a single dose of oral azithromycin (1.0 g) or oral clarithromycin (200 mg × 2/day, 7 days) is required in the pregnant women with CT genital infection for the prevention of neonatal CT infection. However, the screening with CT antibody detection tests may increase the both risks of unnecessary antibiotics administration and no antibiotics administration in the women required antibiotics.

Therefore, Japanese obstetricians should perform CT nucleic acid detection tests from the uterine cervix of the pregnant women thoroughly.

## References

[1] Rours GI, Duijts L, Moll HA, Arends LR, de Groot R, Jaddoe VW, Hofman A (2011). Chlamydia trachomatis infection during pregnancy associated with preterm delivery: a population-based prospective cohort study. Eur J Epidemiol.

[2] Remmington JS, Klein JO, Wilson CB, Baker CJ (2006). Infectious diseases of the fetus and newborn infant.

[3] Rours GI, Hammerschlag MR, Van Doornum GJ, Hop WC, de Groot R, Willemse HF, Verbrugh HA (2009). Chlamydia trachomatis respiratory infection in Dutch infants. Arch Dis Child.

[4] Silva MJ, Florencio GL, Gabiatti JR, Amaral RL, Eleuterio Junior J, Goncalves AK (2011). Perinatal morbidity and mortality associated with chlamydial infection: a meta-analysis study. Braz J Infect Dis.

[5] Rours IG, Hammerschlag MR, Ott A, De Faber TJ, Verbrugh HA, de Groot R, Verkooyen RP (2008). Chlamydia trachomatis as a cause of neonatal conjunctivitis in Dutch infants. Pediatrics.

[6] Pereboom MT, Spelten ER, Mannien J, Rours GI, Morre SA, Schellevis FG, Hutton EK (2014). Knowledge and acceptability of Chlamydia trachomatis screening among pregnant women and their partners; a cross-sectional study. BMC Public Health.

[7] Minakami H, Maeda T, Fujii T, Hamada H, Iitsuka Y, Itakura A, Itoh H (2014). Guidelines for obstetrical practice in Japan: Japan Society of Obstetrics and Gynecology (JSOG) and Japan Association of Obstetricians and Gynecologists (JAOG) 2014 edition. J Obstet Gynaecol Res.

[8] Norman JE, Wu O, Twaddle S, Macmillan S, McMillan L, Templeton A, McKenzie H (2004). An evaluation of economics and acceptability of screening for Chlamydia trachomatis infection, in women attending antenatal, abortion, colposcopy and family planning clinics in Scotland, UK. BJOG.

[9] Cheney K, Wray L (2008). Chlamydia and associated factors in an under 20s antenatal population. Aust N Z J Obstet Gynaecol.

[10] Chen MY, Fairley CK, De Guingand D, Hocking J, Tabrizi S, Wallace EM, Grover S (2009). Screening pregnant women for chlamydia: what are the predictors of infection?. Sex Transm Infect.

[11] Silveira MF, Ghanem KG, Erbelding EJ, Burke AE, Johnson HL, Singh RH, Zenilman JM (2009). Chlamydia trachomatis infection during pregnancy and the risk of preterm birth: a case-control study. Int J STD AIDS.

[12] Moscicki AB, Winkler B, Irwin CE, Jr., Schachter J (1989). Differences in biologic maturation, sexual behavior, and sexually transmitted disease between adolescents with and without cervical intraepithelial neoplasia. J Pediatr.

[13] Smith AM, Rissel CE, Richters J, Grulich AE, de Visser RO (2003). Sex in Australia: the rationale and methods of the Australian Study of Health and Relationships. Aust N Z J Public Health.

[14] Weill FX, Le Hello S, Clerc M, Scribans C, de Barbeyrac B (2010). Serological reactivity and bacterial genotypes in Chlamydia trachomatis urogenital infections in Guadeloupe, French West Indies. Sex Transm Infect.

[15] Nishii O (2010). Chlamydia infection (in Japanese). Obstet Gynecol Practice.

[16] Ozaki K, Shimada M, Nakata M, Fuse Y, Shibata Y, Miyake H, Suzuki S (2013). Is serum antibody detection suitable for screening of genital Chlamydia infection during pregnancy (in Japanese)?. Tokyo Sanka Fujinka Zasshi.

